# The antihypertensive effect of irbesartan in spontaneously hypertensive rats is associated with improvement of the leptin–adiponectin imbalance

**DOI:** 10.1080/21623945.2021.1886409

**Published:** 2021-02-11

**Authors:** Junting Weng, Min Chen, Rongjie Guo, Shuzhen Yang, Danjuan Liu, Dexiang Fang

**Affiliations:** Department of Critical Care Medicine, The Affiliated Hospital of Putian University, Putian, China

**Keywords:** Irbesartan, spontaneously hypertensive rat, leptin, adiponectin, homa-IR

## Abstract

Objective: This study aimed to investigate whether the antihypertensive effect of irbesartan (IRB) in spontaneously hypertensive rats (SHR) was achieved through improvement of insulin resistance and adjustment of the LPN–APN imbalance.Methods:SHR rats were divided into SHAM, SHR-A and SHR-I group(8 per group). Homologous Wistar–Kyoto (WKY) rats were used as control group (WKY).The SHR-I group received 30 mg/kg/d IRB, the SHR-A group received 2.5 mg/kg AML. After 8 weeks, systolic blood pressure (SBP) was measured. The concentrations of blood glucose, insulin, LPN and APN were detected. Rat epididymal adipose tissues were collected to analyze the mRNA expression levels ofepididymal LPN and APN using reverse transcription–polymerase chain reaction. In addition, the LPN/APN ratio was calculated. Results:SBP, homeostasis model assessment of insulin resistance (HOMA-IR), LPN concentration, adipose LPN mRNA expression level, and the LPN/APN ratio increased (*P*<0.05) and APN concentration and adipose APN mRNA expression level decreased (*P*<0.05) in SHR rats.IRB decreased SBP, HOMA-IR, serum LPN, adipose LPN mRNA expression, and the LPN/APN ratio and increased serum APN and adipose APN mRNA expression. Conclusion: The antihypertensive effect of IRB in SHR rats was associated with its improvement of insulin resistance and correction of the LPN–APN imbalance.

**Abbreviations:** ANOVA, one-way analysis of variance; SHR, Spontaneously hypertensive rats; WKY, Wistar kyoto rats; IRB, Irbesartan; AML, Amlodipine; LPN, Leptin; APN, Adiponectin; Ang-II, AngiotensinⅡ; HOMA-IR, Homoeostasis model assessment-insulin resistance; SBP, Systolic blood pressure; RT-PCR, Reverse transcription polymerase chain reaction; ARB, AngiotensinⅡreceptor blocker

## 1. Introduction

Hypertension is a common and frequently occurring disease that seriously endangers human health and is also an important risk factor for cardiovascular diseases. Statistical results showed that nearly 1 billion people in the world have hypertension, of whom 70% have combined cardiovascular diseases. Therefore, prevention and treatment of hypertension have received much attention. Patients with hypertension have leptin–adiponectin (LPN–APN) imbalance [1], elevated LPN levels [2], and decreased APN levels [3].

Adipose tissues not only are an organ for energy storage but also are an endocrine organ for synthesis and secretion of many hormones and cytokines. LPN and APN are both adipogenic endocrine polypeptide hormones secreted by adipocytes. By increasing sympathetic excitability [4], LPN decreases the vasodilation function and nitric oxide (NO) level of endothelial cells [5] and promotes smooth muscle proliferation [6] to influence the development of hypertension. APN not only promotes the catabolism of glucose and lipids but also has anti-inflammatory and anti-atherosclerotic functions and induces the activation of endothelial nitric oxide synthase in smooth muscle endothelial cells [7]. In these ways, it can reduce blood pressure. Adipose tissues have a complete renin-angiotensin-aldosterone system (RAAS). Angiotensin II (Ang-II) can induce adipocytes to synthesize LPN and inhibit adipocytes from secreting APN [8]. It is worth studying whether Ang-II receptor blockers (ARBs) can adjust the LPN–APN imbalance.

Irbesartan (IRB) is an oral ARB. It selectively blocks the interaction between Ang-II and its type 1 (AT1) receptor to inhibit the function of Ang-II, block the RAAS system, and improve insulin sensitivity. Therefore, it is becoming more widely applied in cardiovascular diseases.

In this study, we hypothesized that the antihypertensive effect of IRB in spontaneously hypertensive (SHR) rats was achieved through improvement of insulin resistance and adjustment of the LPN–APN imbalance.

## 2. Material and methods

### 2.1 Rats

A total of 24 male SHR rats (aged 12 weeks) with a body weight of 250 ± 20 g were selected. In addition, there were eight homologous male Wistar–Kyoto (WKY) rats (aged 12 weeks) with a body weight of 250 ± 20 g. Animals were provided by Beijing Vital River Laboratory Animal Technologies Co., Ltd., China [animal licence number: SCXK (Beijing) 2015–0003]. Body weights of animals did not significantly differ. Four animals were housed per cage and fed standard rat food. Animal had access to food and water *ad libitum*. The housing temperature was 20–22°C, the relative humidity was 40%-70%, and the environment was quiet.

### 2.2 Material

The blood glucose reagent kit and the insulin reagent kit were purchased from Beckman (USA). The APN and LPN reagent kits were purchased from Shanghai Xinfan Biotechnology Co., Ltd., China (APN catalogue number:2,015,032,004; LPN catalogue number:2,015,051,009) . The RT-PCR reagent kit was purchased from Shanghai Lanchuang Biotechnology Development Co., Ltd., China. Primers were purchased from Shanghai Biochemical Sci-tech. IRB was purchased from Hangzhou Sanofi-Aventis Minsheng Pharmaceuticals. Amlodipine (AML) was purchased from Pfizer (USA).

### 2.4 Methods

#### 2.4.1 Experimental grouping

A total of 24 male SHR rats (aged 12 weeks) with a body weight of 250 ± 20 g were divided into the sham surgery group (SHAM), AML group (SHR-A), and IRB group (SHR-I), with eight animals in each group. Eight homologous male WKY rats (aged 12 weeks) with a body weight of 250 ± 20 g were used as the normal control group (WKY).

#### 2.4.2 Treatment methods

SHR-I group: 30 mg/kg IRB was dissolved in 1 ml distilled water for gavage. SHR-A group: 2.5 mg/kg AML was dissolved in 1 ml distilled water for gavage. The SHAM group and WKY group received 1 ml distilled water by gavage. They all received gavage once every day for a total of 8 weeks.

### 2.5 Observation indicators and detection methods

#### 2.5.1 Measurement of systolic blood pressure (SBP)

The MRB-IIIA rat sphygmomanometer was used in this experiment. SBP of the tail artery was measured three times in close succession in a quiet room while the rat was awake at the end of 8 weeks. The average value was taken for analysis.

#### 2.5.2 Measurement of serum APN and LPN concentrations

After 8 weeks of the experiment, 2 ml of carotid artery blood of rats was collected, and the serum was separated. The radioimmunoassay was performed according to the manual of the reagent kit. A γ-radiometer was used for counting. Serum concentrations of APN and LPN were calculated.

#### 2.5.3 Measurement of homoeostasis model assessment of insulin resistance (HOMA-IR)

After 8 weeks of the experiment, HOMA-IR was assessed [9]: HOMA-IR = fasting blood glucose (mmol/L) × fasting insulin level (mU/L)/22.5. Blood glucose was measured using the Beckman automatic biochemical analyser (USA). Insulin was measured using the Beckman chemiluminescence analyser (USA). The measurement was performed strictly according to the operational procedures.

#### 2.5.4 Detection of the mRNA expression levels of LPN and APN using RT-PCR

*RNA isolation and cDNA reverse transcription*. After 8 weeks of the experiment, 100 mg of rat epididymal adipose tissue were preserved in TRIzol® reagent (Invitrogen; Thermo Fisher Scientific Inc.) at −80°C until use. Total RNA isolation was performed by following the TRIzol® manufacturer’s protocol with an RNeasy Mini kit (Qiagen, Inc.) for RNA purification. DNase I. SuperScript III First-Strand Synthesis SuperMix (Thermo Fisher Scientific, Inc.) was used for reverse transcription-quantitative PCR (RT-qPCR) to produce cDNA from 2 μg total RNA. The reaction protocol consisted of 25°C for 5 min, 42°C for 60 min and 70°C for 15 min.

*RT-qPCR*. Total RNA was extracted using TRIzol® reagent (Invitrogen; Thermo Fisher Scientific, Inc.) according to the manufacturer’s protocol. Subsequently, the selected results from RNA-sequencing (RNA-seq) were verified by RT-qPCR analysis. cDNA was produced using a Prime Script™ RT-qPCR kit (Takara Bio, Inc.) according to the manufacturer’s protocol. qPCR was performed using SYBR® Premix Ex Taq™ (Takara Bio, Inc.) on a 7900HT fast RT-qPCR instrument (Applied Biosystems; Thermo Fisher Scientific, Inc.) with technical triplicates. Glyceraldehyde-3-phosphate dehydrogenase (GAPDH) was the internal control. The primer sequences were GAPDH: upstream 5ʹ-GTCGGTGTCAACGGATTTG-3ʹ and downstream 5ʹ-GGGTTTCCCATTGATGACC-3ʹ; LPN: upstream 5ʹ-CCAGGATGACACCAAAACCCTC-3ʹ and downstream 5ʹ-ATCCAGGCTCTCTGGCTTCTGC-3ʹ; APN: upstream 5ʹ-CTCCACCCAAGGAAACTTGT-3ʹ and downstream 5ʹ -CTG2GTCCACATTTTTTTCCT-3ʹ.

Thermocycling conditions were as follows: Initial denaturation at 95°C for 5 min, followed with 35 cycles at 95°C for 30 sec and 60°C for 30 sec. The PCR products were 204, 316, and 500 bp, respectively. Products were subject to 2% agarose gel electrophoresis. The grey density values of samples were measured using the Kodak 440CF imaging system and 1D image analysis software. Semiquantitative analyses of mRNA expression levels of LPN and APN were detected compared with the internal control .

#### 2.5.5 Calculation of the LPN/APN ratio

LPN/APN ratio = LPN mRNA expression level/APN mRNA expression level.

### 2.6 Statistical evaluation

The results are represented as the mean ± SD. Differences between all groups were assessed through one-way analysis of variance (ANOVA) followed by Tukey’s post hoc test. *P* < 0.05 was considered statistically significant. All statistical assessments were performed in GraphPad Prism software version 8.0 (GraphPad Software, Inc., San Diego, CA, USA).

## 3. Results

### 3.1 Effects of IRB and AML on SBP of SHR rats

**Figure 1.**
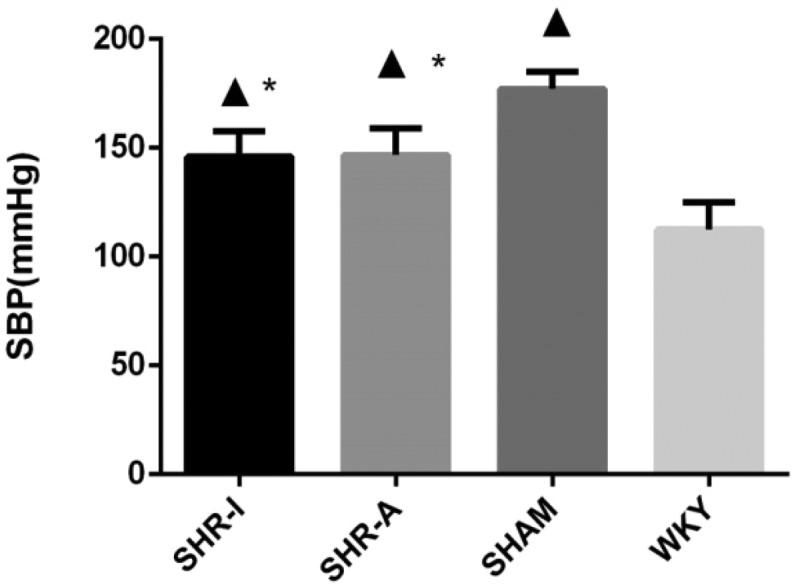
**Effects of IRB and AML on SBP of SHR rats**. SBP was measured in the rat tail artery with the MRB-IIIA rat sphygmomanometer. The results are expressed as mean ± standard deviation (n = 8). ▲ *P* < 0.05 compared to the WKY group; * *P* < 0.05 compared to the SHAM group. IRB = irbesartan. AML = amlodipine

After 8 weeks of the experiment, SBP in the SHAM group was significantly higher than in the WKY group (*P* < 0.05). SBP in the SHR-I group and SHR-A group was lower than in the SHAM group (*P* < 0.05) but was still higher than in the WKY group (*P* < 0.05) (figure 1).

### 3.2 Effect of IRB on HOMA-IR of SHR rats

**Figure 2.**
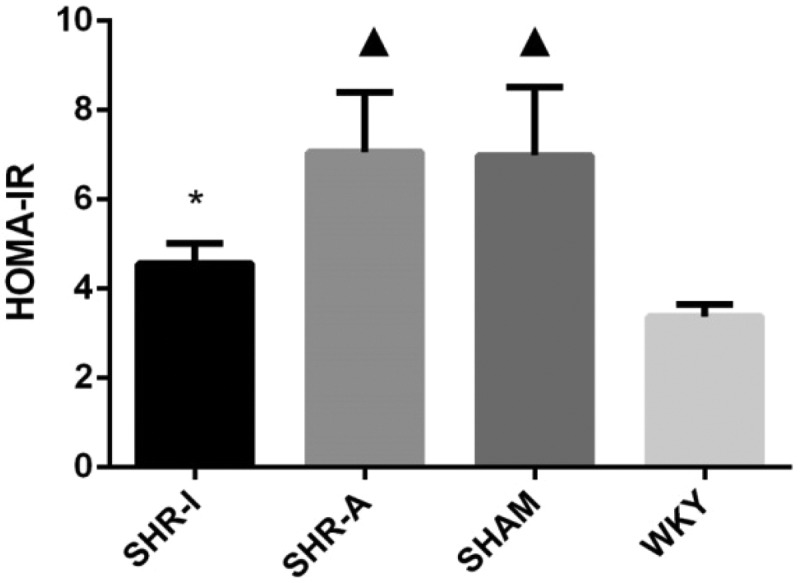
**Effect of IRB on HOMA-IR in SHR rats**. HOMA-IR = fasting blood glucose (mmol/L) × fasting insulin level (mU/L)/22.5. The results are expressed as mean ± standard deviation (n = 8). ▲ *P* < 0.05 compared to the WKY group; * *P* < 0.05 compared to the SHAM group, SHR-A group, and WKY group. IRB = irbesartan

When we detected the effect of IRB on HOMA-IR, we found that HOMA-IR in the SHAM group was significantly higher than in the WKY group (*P* < 0.05). After 8 weeks of the experiment, HOMA-IR in the SHR-I group was lower than in the SHR-A group and SHAM group (*P* < 0.05) but was still higher than in the WKY group (*P* < 0.05) (figure 2).

### 3.3 Effect of IRB on serum LPN concentration and adipose LPN mRNA expression level in SHR rats

**Figure 3.**
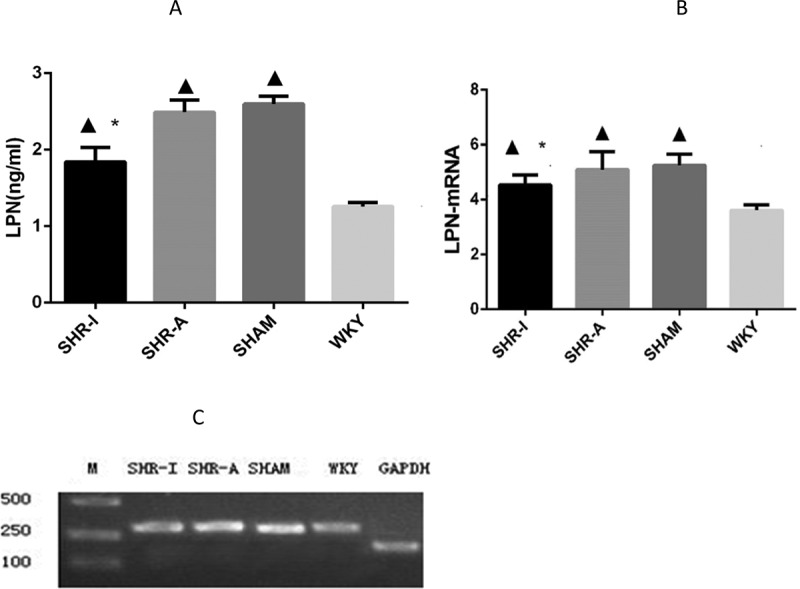
**Effect of IRB on serum LPN concentration and adipose LPN mRNA expression level in SHR rats**. (a)Serum LPN was measured by radioimmunoassay. The results are expressed as mean ± standard deviation (n = 8). ▲ *P* < 0.05 compared to the WKY group; * *P* < 0.05 compared to the SHAM group and SHR-A group. IRB = irbesartan.(b)Adipose LPN mRNA was detected by RT-PCR. The results are expressed as mean ± standard deviation (n = 8). ▲ *P* < 0.05 compared to the WKY group; * *P* < 0.05 compared to the SHAM group and SHR-A group. IRB = irbesartan. (c)Adipose LPN mRNA was detected by RT-PCR. GAPDH was used as the internal control. GAPDH: 204 bp; LPN: 316 bp. The adipose LPN mRNA expression level in the SHAM group was significantly higher than in the WKY group, while the adipose LPN mRNA expression level in the SHR-I group was lower than in the SHAM group and SHR-A group. IRB = irbesartan. M = marker

After 8 weeks of the experiment, the serum LPN concentration in the SHAM group was significantly higher than in the WKY group (*P* < 0.05), whereas the serum LPN concentration in the SHR-I group was significantly lower than in the SHAM group and SHR-A group (*P* < 0.05) (figure 3a). The adipose LPN mRNA expression level in the SHAM group was significantly higher than in the WKY group (*P* < 0.05), whereas the adipose LPN mRNA expression level in the SHR-I group was significantly lower than in the SHAM group and SHR-A group (*P* < 0.05) (figures 3b and 3c).

### 3.4 Effect of IRB on serum APN concentration and adipose APN mRNA expression level in SHR rats

**Figure 4.**
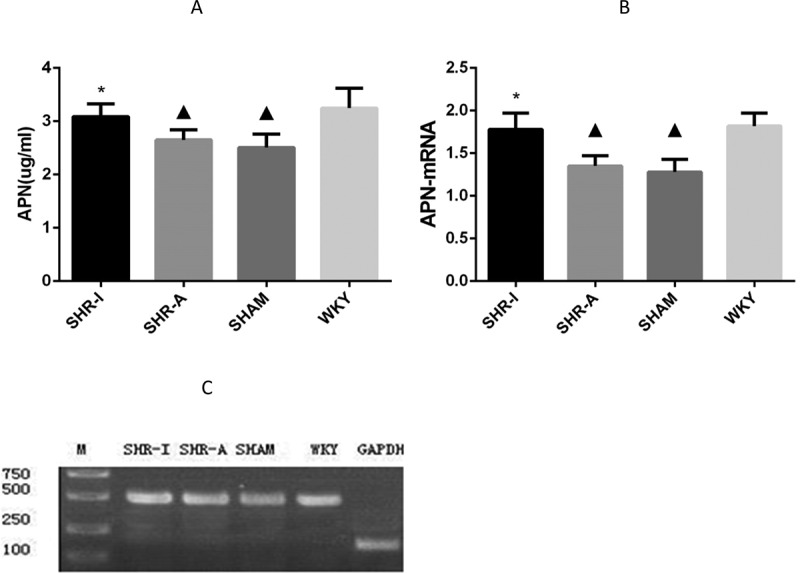
**Effect of IRB on serum APN concentration and adipose APN mRNA expression level in SHR rats**. (a)Serum APN was measured by radioimmunoassay. The results are expressed as mean ± standard deviation (n = 8). ▲ *P* < 0.05 compared to the WKY group; * *P* < 0.05 compared to the SHAM group and SHR-A group. IRB = irbesartan.(b)The results are expressed as mean ± standard deviation (n = 8). ^▲^ *P* < 0.01 compared to the WKY group; * *P* < 0.05 compared to the SHAM group and SHR-A group. IRB = irbesartan.(c)Adipose APN mRNA was detected by RT-PCR. GAPDH was used as the internal control. GAPDH: 204 bp; APN: 500 bp. The adipose APN mRNA expression level in the SHAM group was significantly lower than in the WKY group, while the adipose APN mRNA expression level in the SHR-I group was higher than in the SHAM group and SHR-A group. IRB = irbesartan. M = marker

After 8 weeks of the experiment, the serum concentration and adipose mRNA expression of APN in the SHAM group were significantly lower than in the WKY group (*P* < 0.05), whereas the serum concentration and adipose mRNA expression of APN in the SHR-I group were significantly higher than in the SHAM group and SHR-A group (*P* < 0.05) (figures 4a, 4b, and 4c).

### 3.5 Effect of IRB on the LPN/APN ratio in SHR rats

**Figure 5.**
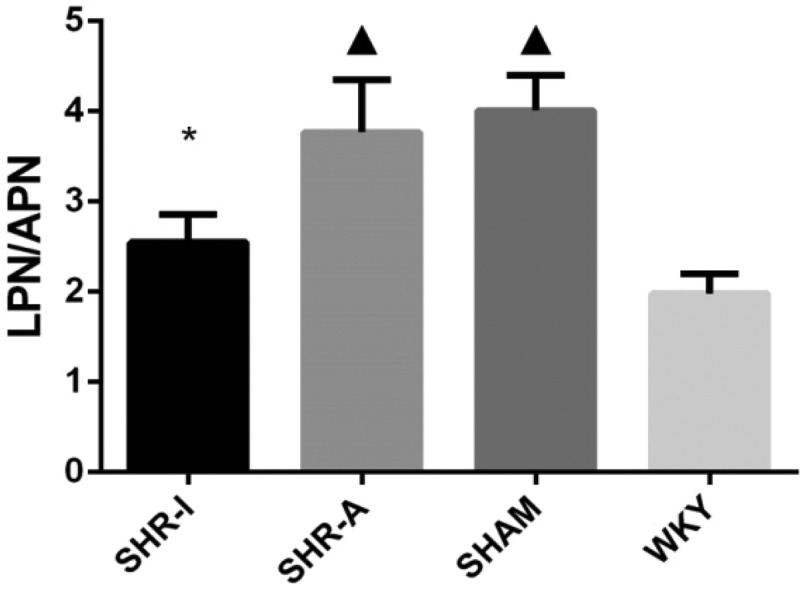
**Effect of IRB on the LPN/APN ratio in SHR rats**. LPN/APN ratio = LPN mRNA expression level/APN mRNA expression level. The results are expressed as mean ± standard deviation (n = 8). ▲ *P* < 0.05 compared to the WKY group; * *P* < 0.05 compared to the SHAM group and SHR-A group. IRB = irbesartan

After 8 weeks of the experiment, the LPN/APN ratio in the SHAM group was significantly higher than in the WKY group (*P* < 0.05), whereas the LPN/APN ratio in the SHR-I group was significantly lower than in the SHAM group and SHR-A group (*P* < 0.05) (figure 5).

## 4. Discussion

LPN and APN are both endocrine polypeptide hormones secreted by white adipose tissues. LPN is a protein of 167 amino acids (aa). After secretion into blood, the N-terminal 21 aa signal peptide will be removed to form active LPN, which contains 146 aa and has the relative molecular weight of 16 kD. LPN is a risk factor for cardiovascular diseases [10]. Human APN is 244 aa (mouse APN is 247 aa) including the N-terminal signal peptide, the N-terminal nonhelical functional region, the collagen domain, and the C-terminal globular domain. After posttranslational modification, there are eight different homologous APN proteins that are protective factors against cardiovascular diseases [11].

The causes of hypertension are multifactorial. Insulin resistance is considered one of the pathophysiological bases of development of hypertension [12]. It is worth studying whether insulin resistance affects hypertension development through LPN–APN imbalance. This study found that HOMA-IR, serum LPN concentration, and adipose LPN mRNA expression in rats in the SHAM group were higher than in the WKY group, while serum APN concentration and adipose APN mRNA expression in rats in the SHAM group were lower than in the WKY group, and the LPN/APN ratio in the SHAM group was significantly higher than in the WKY group. These results indicate that hypertension involved a LPN–APN imbalance, increased LPN, and decreased APN.

There is clinical evidence of improvement of insulin resistance by ARBs. The results of two large-scale studies, the Losartan Intervention For Endpoint reduction in hypertension study (LIFE) [13] and the Valsartan Antihypertensive Long-term Use Evaluation (VALUE) trial [14], showed that ARBs were effective at improving insulin resistance. This study showed that, after IRB treatment for 8 weeks, the HOMA-IR value in the SHR-I group significantly decreased compared to that in the SHAM group. There are a few possible mechanisms underlying the improvement of insulin resistance by RAAS system blockers [15]: ① Ang-II can increase serine phosphorylation of insulin receptor substrate-1 (IRS-1) and decrease the activity of IRS-1-associated phosphoinositide 3-kinase (PI3K), hindering insulin signal transduction, whereas RAAS blockers reverse this effect. ② By blocking the AT1 receptor, ARBs improve endothelial functions, inhibit oxidative stress, exert anti-inflammatory effects, and increase tissue sensitivity to insulin. ③ Ang-II inhibits lipid formation, so excessive triglycerides cannot be stored in lipids and get ectopically deposited in insulin-sensitive tissues such as liver and muscle to promote the development of insulin resistance.

Studies in recent years [8] showed that human and mouse adipose tissues have a complete RAAS system, and Ang-II can activate LPN mRNA expression in adipocytes to inhibit APN mRNA expression in adipocytes.

Cassis et al [16] reported that Ang-II stimulated adipocytes to synthesize and secrete LPN, whereas decreasing or blocking Ang-II synthesis and function by ARBs inhibited LPN synthesis and secretion. We also found that, after IRB treatment, LPN synthesis and secretion in rat adipose tissue were inhibited. LPN is produced by adipose tissues and acts in the hypothalamus to regulate food intake and energy metabolism. Its secretion level is determined by the amount of adipose tissue. Increased lipids also increase LPN synthesis, feeding back to the hypothalamus to inhibit food intake and increase energy metabolism. Lower LPN secretion stimulates food intake and decreases metabolism. The results in this study showed that the serum LPN concentration and LPN mRNA expression level in the IRB group both decreased, suggesting that the RAAS regulates LPN metabolism through specific AT1 receptors and that Ang-II and AT1 receptor antagonists inhibit LPN synthesis and secretion. There is a ‘fat–insulin secretion axis’ in the human body. A bidirectional feedback loop is formed between adipose tissues and islets through LPN, APN, and insulin. Many animal studies have confirmed that insulin can increase LPN secretion in adipose tissues [17]. Under normal conditions, insulin can stimulate adipose tissues to secrete LPN. Increased plasma LPN can transduce signals to obesity gene receptors in the hypothalamus to inhibit neuropeptide Y gene expression to cause food intake reduction and energy consumption and inhibit islet β-cells to secrete insulin, which feeds back to decrease LPN production [18]. Therefore, we can speculate that IRB might decrease the insulin secretion pathway through improvement of insulin resistance to decrease LPN synthesis and secretion.

From many studies on the treatment of hypertension with antihypertensive drugs, it can be speculated that one of their antihypertensive mechanisms is to improve insulin resistance. High insulin can inhibit APN synthesis and secretion. One possible mechanism is that insulin binds to its specific receptor to decrease APN expression through a reduction in IRS-1-associated PI3K activity [19]. Furuhashi et al [15] compared the effects of temocapril and candesartan on APN. The increase in APN caused by the latter was significantly higher than that by the former, and they significantly decreased the fasting insulin level. The results in this study showed that, after application of IRB for 8 weeks, the HOMA-IR value decreased, and the serum APN concentration and adipose APN mRNA expression level increased. Therefore, we speculate that ARB decreased the inhibitory effect of high insulin on APN synthesis and secretion through the improvement of insulin sensitivity to promote APN synthesis and secretion.

The LPN/APN ratio is an indicator reflecting disorders of adipose tissue function [20]. The association of the LPN/APN ratio with insulin resistance is stronger than that of LPN, APN, or HOMA-IR [21]. Our study indicated that SHR rats had LPN–APN imbalance and a higher LPN/APN ratio. After application of IRB, the LPN/APN ratio significantly decreased, indicating that IRB could improve the LPN–APN imbalance in SHR rats.

This study showed that after SHR rats were treated with IRB or AML for 8 weeks, blood pressure was similarly reduced in these two groups. IRB improved insulin sensitivity to reduce the serum LPN concentration and adipose LPN mRNA expression, increase the serum APN concentration and adipose APN mRNA expression, decrease the LPN/APN ratio, and adjust the LPN–APN imbalance. AML did not have any of the above effects. These results indicate that not all antihypertensive drugs have the same effects. The effect of IRB on LPN and APN was not achieved through reduction of blood pressure. In addition, the Ang-II receptor antagonist IRB exerted its antihypertensive function by adjusting the LPN–APN imbalance.

## 5. Conclusion

The antihypertensive effect of IRB in SHR rats was associated with improvement of insulin resistance and adjustment of the LPN–APN imbalance. Because LPN is a risk factor for cardiovascular diseases and APN is a protective factor against cardiovascular diseases, application of IRB to adjust the LPN–APN imbalance is a possible antihypertensive treatment that could reduce the development of cardiovascular events in hypertensive patients. Therefore, it is worth promoting in the clinic.

## Highlights

The antihypertensive effect of IRB in SHR rats is associated with improvement of insulin resistance. The antihypertensive effect of IRB in SHR rats is associated with correction of the LPN–APN imbalance.
